# A Theoretical Study on the Structural, Electronic, and Magnetic Properties of Bimetallic Pt_13−n_Ni_n_ (N = 0, 3, 6, 9, 13) Nanoclusters to Unveil the Catalytic Mechanisms for the Water-Gas Shift Reaction

**DOI:** 10.3389/fchem.2022.852196

**Published:** 2022-03-25

**Authors:** Manoel Victor Frutuoso Barrionuevo, Juan Andrés, Miguel Angel San-Miguel

**Affiliations:** ^1^ UNICAMP Materials Simulation Lab, Institute of Chemistry, Department of Physical-Chemistry, University of Campinas, Campinas, Brazil; ^2^ Química Teórica y Computacional, Department de Química Física i Analítica, Universitat Jaume I, Castellón de la Plana, Spain

**Keywords:** water-gas shift reaction, bimetallic (Pt/Ni) nanoclusters, DFT, density functional theory, heterogeneous catalysis, first-principles calculations

## Abstract

In this work, first-principles calculations by using density functional theory at the GFN-xTB level, are performed to investigate the relative stability and structural, electronic, and magnetic properties of bimetallic Pt_13−n_Ni_n_ (n = 0, 3, 6, 9, 13) nanoclusters by using corrected Hammer and Nørskov model. In addition, by employing the reaction path and the energetic span models, the energy profile and the turnover frequency are calculated to disclose the corresponding reaction mechanism of the water-gas shift reaction catalyzed by these nanoclusters. Our findings render that Ni causes an overall shrinking of the nanocluster’s size and misalignment of the spin channels, increasing the magnetic nature of the nanoclusters. Pt_7_Ni_6_ nanocluster is the most stable as a result of the better coupling between the Pt and Ni *d*-states. Pt_4_Ni_9_ maintains its structure over the reaction cycle, with a larger turnover frequency value than Pt_7_Ni_6_. On the other hand, despite Pt_10_Ni_3_ presenting the highest value of turnover frequency, it suffers a strong structural deformation over the completion of a reaction cycle, indicating that the catalytic activity can be altered.

## 1 Introduction

Catalysis plays a pivotal role in all strategies for establishing energy- and atom-efficient sustainable chemical technologies. One of the green chemistry goals is to find alternatives for the production of green fuels that either do not contribute to the increase of atmospheric CO_2_ concentration or reuse CO_2_ in the industrial chain. Hydrogen gas, H_2_(g), is a storable and green form of chemical energy with many practical applications (Zou and Zhang, 2015). The water-gas shift (WGS) reaction, CO(g) + H_2_O(g) → CO_2_(g) + H_2_(g), provides an important process for the H_2_(g) production (Yao et al., 2017), and also it is the main path for sequestrating CO(g), a toxic gas responsible for causing death from asphyxiation (Blumenthal, 2001). Nevertheless, the literature suggests that WGS can be employed for carbon-capturing, aiming to produce acetic acid from activated CO_2_(g) (Somiari and Manousiouthakis, 2017). To reach high reaction activity, the design of efficient catalysts is important (Beller et al., 2010; Jeong et al., 2013; Lin et al., 2013; Roşca et al., 2015; Zhang et al., 2016).

**FIGURE 1:**
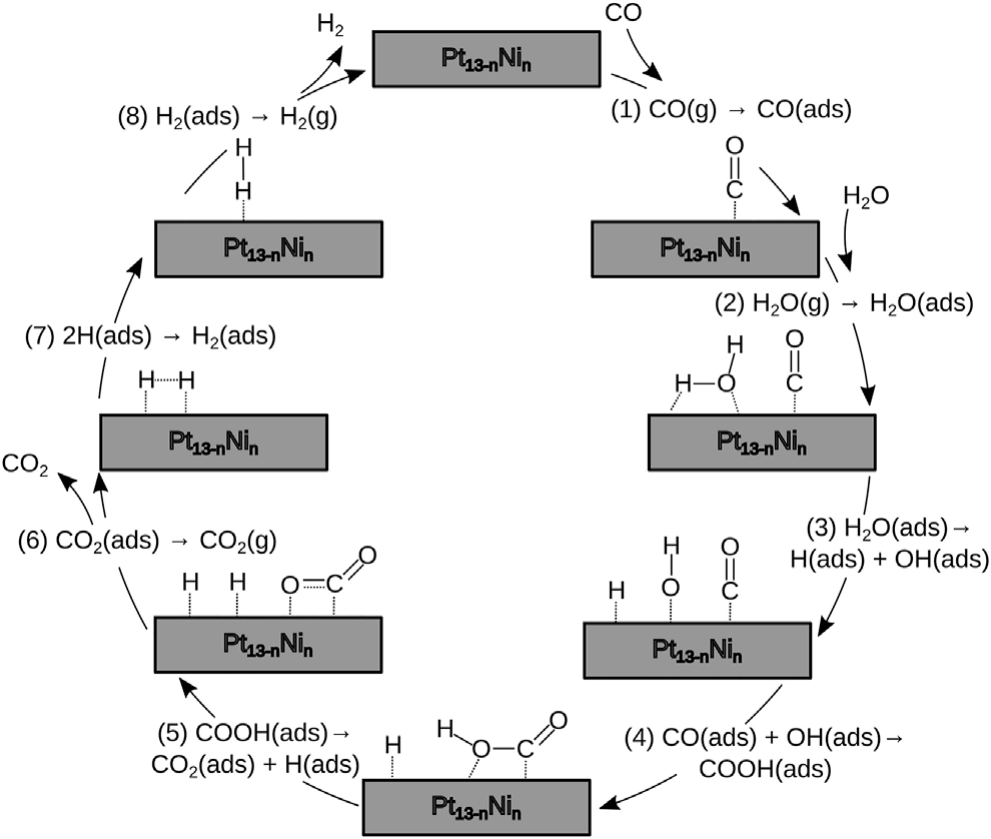
Associative carboxylate mechanism for the WGS reaction taking place over the nanocluster’s surface. The eight steps correspond to Eqs 1–8.

Heterogeneous catalytic processes generally include reactant molecular adsorption, dissociated atom reactions at the catalyst surface, and product molecular desorption. In particular, the reaction mechanism of the WGS reaction over metal surfaces might occur at least along eight steps to turn a complete cycle over the surface of the metal catalyst (Eqs 1–8), in which CO would be chemically activated first once it is adsorbed to the catalyst surface, followed by the H_2_O adsorption. Only then, the water hydrolysis would take place *via* an associative carboxylate mechanism, as shown in Figure 1 (Lian et al., 2015; Baraj et al., 2021).

CO(gas)→CO(ads) (1)

$$H_{2}O\left(\text{gas}\right)\to H_{2}O\left(\text{ads}\right)$$ (2)

$$H_{2}O\left(\text{ads}\right)\to OH\left(\text{ads}\right)+H\left(\text{ads}\right)$$ (3)

CO(ads)+OH(ads)→COOH(ads) (4)

$$COOH\left(\text{ads}\right)\to CO_{2}\left(\text{ads}\right)+H\left(\text{ads}\right)$$ (5)

$$CO_{2}\left(\text{ads}\right)\to CO_{2}\left(\text{gas}\right)$$ (6)

$$H\left(\text{ads}\right)+H\left(\text{ads}\right)\to H_{2}\left(\text{ads}\right)$$ (7)

$$H_{2}\left(\text{ads}\right)\to H_{2}\left(\text{gas}\right)$$ (8)

Designing new catalysts and understanding how they work is a time and resource-consuming process, necessitating an increasing reliance on tools that can predict catalyst performance. Computational techniques mainly employing density functional theory (DFT) methods have been used to study the structure, composition, and properties for the design of new catalysts based on metal alloys (Greeley, 2016). From a theoretical point of view, the *d*-band center model of Hammer and Nørskov (HN), developed more than 2 decades ago, is widely used for understanding and predicting catalytic activity on transition metal surfaces (Hammer and Nørskov, 1995, 2000; Hammer et al., 1996; Greeley et al., 2002; Xin et al., 2014) and offers a way to find a relationship between the catalytic activity and the characteristics of the electronic *d*-band profile (Morikawa et al., 1997). In the HN model, the center of the band of the *d*-states participating in the interaction between the surface atoms and an adsorbate molecule is approximated with a single state at energy *ϵ*_*d*_. Such a model can be understood as a narrow *d*-band limit of the Newns-Anderson model (Anderson, 1961; Newns, 1969), and then suggests how prone the material is to work as a catalyst (Ínoğlu and Kitchin, 2010). The variation of the adsorption energy from one transition metal surface to another might be associated to the upward shift of *ϵ*_*d*_ with respect to the Fermi energy, i.e., the closer *ϵ*_*d*_ is to the Fermi level, the stronger the adsorption might be. However, studies have shown that relying solely on *ϵ*_*d*_ may be misleading; then, the *d*-band as a whole is used by calculating the *d*-bandwidth $\left(\bar{w}\right)$ (Xin et al., 2014). Moreover, there is also a discussion that *ϵ*_*d*_ might be subjected to the polarization imposed by differences within the spin-channel occupation for open-shell systems (Bhattacharjee et al., 2016). Therefore, for the analysis of *ϵ*_*d*_, it is convenient to observe both $\bar{w}$ and its spin-channel occupancy.

Another way for assessing the performance of a catalyst is by obtaining its turnover frequency (TOF) by means of the energetic span (ES) model to calculate the energy profile of a reaction mechanism (Christiansen, 1953; Kozuch and Shaik, 2006, 2008; Uhe et al., 2010; Solel et al., 2019). Each reaction step might offer a degree of control for the entire mechanism, which can be used to identify the rate-determining step, known as X_TOF_. If the X_TOF_ value belongs to an intermediate state, then it is called X_TOF_ of a putative TOF determining intermediate (TDI, X_TDI_). On the other hand, if that belongs to a transition state, it is labeled as X_TOF_ of a putative TOF determining transition state (TDTS, X_TDTS_). The values of both X_TDI_, and X_TDTS_ range from 0 to 1, and the larger the value, the more important the corresponding stage is for the reaction mechanism. Thus, the TOF can be computed once the X_TDI_ and X_TDTS_ values are calculated. This strategy is applied for different reactions in Kozuch’s papers (Kozuch and Shaik, 2006; Kozuch and Shaik, 2008; Uhe et al., 2010; Solel et al., 2019). One key aspect of the ES model that differentiates it from the simple HN model is the possibility of assessing the temperature effects over the catalyzed mechanism. Such an approximation might help better describe the behavior of nanoclusters working as catalysts.

Metal nanoalloys, which are nanostructures consisting of one or more metal species, have received increased attention as their properties can be tuned by varying their composition and size. Nanoalloys exhibit a broad array of chemical ordering patterns depending on the interaction between the constituent metals. Then, they are attractive from a fundamental perspective and often show distinct catalytic properties and stability as compared to individual metals, which is typically ascribed to the coupling effect and/or synergism in the electronic and/or geometric structures that occur when the metals are combined (Ferrando et al., 2008; Tao, 2012; Yan et al., 2017; Ding et al., 2018; Añez et al., 2021; Reboul et al., 2021). Significant efforts have been put into the research field of nanocatalysis to explore the structure, surface composition, and atomic distribution for two metals systems, which are crucial for their properties and catalytic activities of bimetallic nanoalloys (Ferrando et al., 2008; Jellinek, 2008; Piotrowski et al., 2012; Cui et al., 2013; Zhao et al., 2013; Guedes-Sobrinho et al., 2015; Gilroy et al., 2016; Wang et al., 2016; Wu et al., 2019). However, an in-depth understanding of the processes involved in forming these alloy materials is somewhat limited, especially at the atomic level.

From a theoretical point of view, studies have demonstrated that dispersion effects play an important role in the adsorption energy (Davis et al., 2015), leading to subtle changes in the adsorption process, particularly for Pt nanoclusters, which could modify the energetic description of the mechanism. Since the proposal of dispersion correction models such as the Grimme’s family of corrections (Grimme, 2004), many studies have been conducted to investigate the influence of such a description in different systems, from discrete organic molecules to periodic solid materials (Barone et al., 2009; Davis et al., 2015; Kovács et al., 2017). Those works mostly pointed out improvements in computational results when employing generalized gradient approximation (GGA) type functionals allied with dispersion corrections. Recently, Grimme et al. have developed a tight-binding (TB) method parameterized for most elements of the periodic table (Z = 1–86) entitled GFN-xTB (Grimme et al., 2017; Grimme, 2019). It allows a fast and accurate prediction of chemical structures for a variety of species, organic or inorganic materials, and even solid materials of periodic nature, which may offer an alternative path for exploratory analysis of new materials for a broad range of applications including catalysis. Nevertheless, the GFN-xTB presents a set of tools for quick assessment of reaction mechanisms, known as the reaction path (RP) model. The RP model works as a Gaussian biasing potential for both pushing the reactant towards the products and pulling the products towards the reactants (Grimme et al., 2019; Rasmussen and Jensen, 2020).

Previous studies render that both Pt and Ni pure systems are suitable catalysts for WGS reaction, but the former is prone to CO poisoning, and the latter tends to favor a methanation reaction pathway (Senanayake et al., 2011; Alijani and Irankhah, 2013; Ammal and Heyden, 2019; Nakhaei Pour and Tayyari, 2019; Palma et al., 2020). Regarding structural, magnetic, and electronic characteristics of bimetallic Pt_13−n_Ni_n_ (n = 0–13) nanoclusters, some studies have shown that Ni does not provide any significant structural deformation but can weak the nanoalloy binding energy and increase its magnetic nature (Jia et al., 2013; Zhao et al., 2013). Concerning the WGS reaction, theoretical studies of PtNi nanoclusters with different atomic quantities of Pt and Ni have been published, exposing the possibility of using those nanoclusters as catalysts (de Souza Monteiro et al., 2008; Lin et al., 2011; Piotrowski et al., 2012; Zhao et al., 2013; Guedes-Sobrinho et al., 2015; Lian et al., 2015; Roduner and Jensen, 2015; Mokkath, 2018). Yet for the WGS reaction, previously computed adsorption energy values suggested that CO adsorption would occur more strongly than H_2_O on Pt_3_M (M = Cu, Mo, Ni, Rh) nanoclusters (Lian et al., 2015). However, to the best of our knowledge, many of those studies lack a description of the dispersion forces and mainly use small-sized nanoalloys having from 3 to 4 atoms when conducting an entire reaction cycle. Moreover, a full description spanning from the structural, electronic, and magnetic properties to the reaction mechanism of PtNi nanoalloys for the WGS reaction is scarce in the literature for systems with 13 atoms or more.

Thus, the purpose of this work is threefold. First, we aim to provide insights into the geometrical, electronic, and magnetic nature of Pt_13−n_Ni_n_ (n = 0, 3, 6, 9, and 13) nanoclusters based on realistic HN models. The second goal is to rationalize the experimental results and understand the origin of the catalytic performance of these bimetallic nanoalloys for the WGS reaction by using the GFN-xTB method (Eqs 1–8). Finally, the energetic span model has been employed to analyze the influence of each step over the TOF. The results of these calculations can be considered a first-principles guided synthesis for pre-treatments to control the initial surface composition of Pt_13−n_Ni_n_ nanoclusters as a model to explore the reaction mechanism for the WGS reaction. This paper contains three more sections. The next section is devoted to the computational methods and model systems. In section three, the results are presented and discussed. The main conclusions are summarized in the fourth and final section.

## 2 Computational Methods and Model Systems

### 2.1 Structural and Energetic Analysis

As one of the most stable structures of PtNi nanoclusters lies within 13 atoms regime and icosahedral shape (Watzky and Finke, 1997; Kaatz and Bultheel, 2019), we employed the third level of GFN-xTB family for quickly assessing all possible atomic arrangements within the Pt_13−n_Ni_n_ composition (n = 0, 3, 6, 9, and 13). As these nanoclusters are known to have an icosahedral shape it is possible that a simple permutation of all atom positions would lead to symmetrically equivalent configurations, which can be discarded. The generation of all nanocluster structures for different compositions was carried out by an in-house package written in Python. Once the structures were relaxed, their local energy minima were confirmed by computing the hessian at the same theory level. Hence, by employing the Quantum-ESPRESSO (QE) (Giannozzi et al., 2017) package within the generalized gradient approximation (GGA) (Perdew et al., 1992) and using the Perdew-Burke-Ernzerhof (Perdew et al., 1996) functional with D3BJ correction (Grimme et al., 2010; Becke and Johnson, 2005; Johnson and Becke, 2005, 2006), we assessed if at least three samples for each nanocluster composition had a matching energy profile as that found by GFN-xTB (details can be found in the Supplementary Material). Finally, we selected those candidates with the minimal energy value for each composition group.

Concerning the nanocluster structure stability, we computed the binding energy per atom (*E*_b_) as described in Eq. 9 and the excess energy (*E*_exc_) as described in Eq. 10.

$$E_{\text{b}}=\frac{E_{\text{tot}}-\sum_{i}^{m}n_{i}E_{i}}{\sum_{i}^{m}n_{i}}$$ (9)

E_tot_, n_*i*_, and E_*i*_ are the total energy of the nanocluster, the number of times the *i*th atom appears for the *m* elements within the nanocluster formula, and its energy as an isolated atom, respectively.

$$E_{\text{exc}}=\frac{1}{13}\left(E_{\text{tot}}^{{\text{Pt}}_{13-\text{n}}{Ni}_{\text{n}}}-\frac{\text{n}}{13}E_{{\text{Ni}}_{13}}-\frac{13-\text{n}}{13}E_{\text{tot}}^{{\text{Pt}}_{13}}\right)$$ (10)

$E_{\text{tot}}^{{\text{Pt}}_{13-\text{n}}{Ni}_{\text{n}}}$ , $E_{\text{tot}}^{{\text{Pt}}_{13}}$ , and $E_{\text{tot}}^{{\text{Ni}}_{13}}$ are the total energy of the Pt_13−n_Ni_n_, Pt_13_, and Ni_13_ nanoclusters, respectively.

### 2.2 Electronic Analysis

Through the analysis of the projected density of states (PDOS) we assessed the magnetic nature of the nanoclusters, as well as the *d*-band center (*ϵ*_*d*_), and bandwidth $\left(\bar{w}\right)$ values as proposed by Hammer-Nørskov model (Kitchin et al., 2004; Nørskov et al., 2011; Vojvodic et al., 2014) (HN) following Eqs 11, 12.

$$\epsilon_{d}=\frac{\int_{-\infty }^{\infty }n_{d}\left(\epsilon \right)\epsilon \text{d}\epsilon }{\int_{-\infty }^{\infty }n_{d}\left(\epsilon \right)\text{d}\epsilon }$$ (11)

$$\bar{w}=\frac{1}{3}{\left(\frac{1}{0.5-f_{d}}\epsilon_{d}\right)}^{2}\left(1-3f_{d}+3f_{d}^{2}\right)$$ (12)

*ϵ*_*d*_ is taken as the energy average of the *d*-band for the electronically occupied fraction, comprising all the density of states (DOS) from negative infinity to the Fermi level (*ϵ*_*f*_). For $\bar{w}$ the *f*_*d*_ is the fractional electron occupation number of the *d*-band for the metal atom.

To compute *f*_*d*_ one can simply integrate the PDOS of the *d*-band of the metal atom assuming the sum of both spin channels, spin-up (*α*), and spin-down (*β*). Moreover, the HN model also proposed a corrected value (see Eq. 13), which considers the bandwidth to enhance the *d*-band center location with respect to the shifts observed in the bandwidth (Nørskov et al., 2014).

$$\epsilon_{d}^{\bar{w}}=\epsilon_{d}-\frac{1}{2}\bar{w}$$ (13)

However, as already pointed out by previous studies (Bhattacharjee et al., 2016), the HN model suffers from not describing the spin polarization role in lowering the *d*-band center. Hence, an effective *d*-band center $\left(\epsilon_{d}^{eff}\right)$ is computed by taking into account the *d*-band center and fractional occupancy of the spin channel as proposed in Eq. 14:

$$\epsilon_{d}^{eff}=\epsilon_{d}-\left(\epsilon_{d↓}-\epsilon_{d↑}\right)\frac{f_{d↑}-f_{d↓}}{f_{d↑}+f_{d↓}}$$ (14)

*ϵ*_*d↑*_, *ϵ*_*d↓*_, *f*_*d↑*_, *f*_*d↓*_, represent the *d*-band center and fractional occupancy for the up, and down spin channels, respectively.

### 2.3 Reaction Path

The RP model works as a Gaussian biasing potential that pushes the reactants towards the products in a stepwise manner; every pushing step is followed by a pulling potential that forces the products back to the reactants. For each step, an optimization is made in order to minimize the total system energy (E_tot_) as given by Eq. 15.

$${\text{E}}_{\text{tot}}={\text{E}}_{\text{tot}}^{\text{el}}+k_{\text{push}}e^{-\alpha \Delta_{\text{r}}^{2}}+k_{\text{pull}}e^{-\alpha \Delta_{\text{p}}^{2}}$$ (15)

in which ${\text{E}}_{\text{tot}}^{\text{el}}$ , *k*_push_, and *k*_pull_ figure as the total electronic energy, positive and negative force constants, respectively. Those constants are applied to the Gaussian construction of the root-mean-squared deviation (RMSD) of the reactants $\left(\alpha \Delta_{\text{r}}^{2}\right)$ and products $\left(\alpha \Delta_{\text{p}}^{2}\right)$ , in which *α* determines the extension of such a biasing.

Under the description of the RP model, we analyze the different steps, 1–8, taking place over the nanoclusters’ surface for n = 3, 6, and 9. Since the RP model uses biasing parameters, we have optimized those values to perform the reaction path as already suggested in the literature (Rasmussen and Jensen, 2020). Herein, the values of *k*_push_ = 0.01 E_h_, *k*_pull_ = 0.02 E_h_, and *α* = 0.03 Bohr^−1^ were employed for the RP model.

All posible adsorption sites were examined for each explored reaction by employing an in-house program written in Python. The routine was set to identify the best adsorption sites, i.e., which adsorption would lead to the lowest energy. Then, the values of the adsorption energy of CO at different sites (Pt, Ni, and PtNi, PtPt and NiNi bridge, and in a hollow position spanning three atoms at a time as Pt_3_, Pt_2_Ni, or PtNi_2_) for each cluster are calculated.

Once the minimal energy paths were explored through the RP model, we computed the zero-point energy (ZPE) for energy corrections, and the transition structures were assessed by verifying they only presented one imaginary frequency.

### 2.4 Assessing the Catlytic Cycle

In a steady-state regime, the TOF can be computed as the net rate of product formation given by Eq 16 (Kozuch and Shaik, 2006, 2008):

$$\text{TOF}=\frac{e^{-\Delta {\text{E}}_{\text{rx}}}-1}{\sum_{\text{i, j}=1}^{\text{N}}e^{{\text{T}}_{\text{i}}-{\text{I}}_{\text{j}}-\delta {\text{E}}_{\text{i, j}}^{\prime }}}$$ (16)

$$\delta {\text{E}}_{\text{i, j}}^{\prime }=\left\{\begin{array}{cc} \Delta {\text{E}}_{\text{rx}}\; & \text{if i}>j\\ 0\; & \text{if i}\leq j \end{array}\right.$$

the transition state (T_i_), intermediate (I_j_), and reaction energy (ΔE_rx_) are given in k_b_T units, in which a specific temperature value can be set for assessing its effect over the TOF value, as shown by Eq. 17a, Eq. 17b, and Eq. 17c.

$${\text{T}}_{\text{i}}=\text{E}\left({\text{T}}_{\text{i}}\right)/{\text{k}}_{\text{b}}\text{T}$$ (17a)

$${\text{I}}_{\text{j}}=\text{E}\left({\text{I}}_{\text{j}}\right)/{\text{k}}_{\text{b}}\text{T}$$ (17b)

$$\Delta {\text{E}}_{\text{rx}}=\left\{\text{E}\left(\text{products}\right)-\text{E}\left(\text{reactants}\right)\right\}/{\text{k}}_{\text{b}}\text{T}$$ (17c)

where E (T_i_) and E (I_j_) represent the energy of the *i*th and *j*th transition and intermediate states, respectively.

Despite the sum of all states found in a reaction cycle seen in the denominator term of Eq. 16, not all reaction steps contribute equally for the energy landscape of the reaction cycle, i.e., some transition states can be higher in energy than others, as some intermediates might be lower in energy. Therefore, it is possible to compute how each step contributes to the overall reaction energy profile as a transition state (Eq. 18) or intermediate (Eq. 19).

$${\text{X}}_{{\text{TOF,T}}_{\text{i}}}=\frac{\sum_{\text{j}}e^{{\text{T}}_{\text{i}}-{\text{I}}_{\text{j}}-\delta {\text{E}}_{\text{i, j}}^{\prime }}}{\sum_{\text{i, j}}e^{{\text{T}}_{\text{i}}-{\text{I}}_{\text{j}}-\delta {\text{E}}_{\text{i, j}}^{\prime }}}$$ (18)

$${\text{X}}_{{\text{TOF,I}}_{\text{j}}}=\frac{\sum_{\text{i}}e^{{\text{T}}_{\text{i}}-{\text{I}}_{\text{j}}-\delta {\text{E}}_{\text{i, j}}^{\prime }}}{\sum_{\text{i, j}}e^{{\text{T}}_{\text{i}}-{\text{I}}_{\text{j}}-\delta {\text{E}}_{\text{i, j}}^{\prime }}}$$ (19)

Values from 0 to 1 are computed for ${\text{X}}_{{\text{TOF,T}}_{\text{i}}}$ and ${\text{X}}_{{\text{TOF,I}}_{\text{j}}}$ . Thus the TDTS and the TDI are identified for values closer to 1 for ${\text{X}}_{{\text{TOF,T}}_{\text{i}}}$ and ${\text{X}}_{{\text{TOF,I}}_{\text{j}}}$ , respectively. Hence, under the ES model an approximation is proposed (Christiansen, 1953; Kozuch and Shaik, 2006, 2008; Uhe et al., 2010; Solel et al., 2019) as shown by Eq. 20.

$$\text{TOF}\approx e^{-\delta \text{E}}$$ (20)

$$\delta \text{E}=\left\{\begin{array}{cc} {\text{T}}_{\text{TDTS}}-{\text{I}}_{\text{TDI}}\; & \text{if i}>j\\ {\text{T}}_{\text{TDTS}}-{\text{I}}_{\text{TDI}}+\Delta {\text{E}}_{\text{rx}}\; & \text{if i}\leq j \end{array}\right.$$

That approximation can be useful for systems that present few rate-determining states and has been shown to give similar results as Eq. 16 (Kozuch and Shaik, 2006, 2008; Kozuch and Matrin, 2011; Solel et al., 2019).

That being said, we assessed each catalytic cycle by employing the ES model within a range of temperatures varying from 700 to 1000 K, with the temperature step of 5 K. As a final remark to verify the nanocluster’s capability of recovering its initial state, we assessed the RMSD for the nanocluster’s structure in each reaction step. In other words, by aligning only the metal atoms that compose the nanocluster, we verified the structural deformation that each nanocluster underwent after a complete reaction cycle.

The RMSD value was computed as shown by Eq 21, where the sum of the simple euclidean distances of each Cartesian coordinate of the target structure (*β*) with respect to the reference model (*α*) is assessed for each *i* atom. The initial nanocluster structure was chosen as the reference model, which returns an RMSD value of zero for the first step. The higher the RMSD value for the following steps, the greater the nanocluster deformation will be with respect to the initial nanocluster structure.

$$RMSD=\sqrt{\frac{1}{N}\overset{N}{\underset{i=1}{\sum }}\left({\left(\alpha_{ix}-\beta_{ix}\right)}^{2}+{\left(\alpha_{iy}-\beta_{iy}\right)}^{2}+{\left(\alpha_{iz}-\beta_{iz}\right)}^{2}\right)}$$ (21)

## 3 Results

### 3.1 Nanocluster Structures

The first step consisted of generating all unique structures for each nanocluster composition. Then, these structures were relaxed using the GFN-xTB method, and only positive frequencies were found after the optimization step; thus, structures with the lowest energy were selected to proceed to the next stages. A pool of nanocluster candidates was obtained for each composition (see Supplementary Table S1).

**FIGURE 2:**
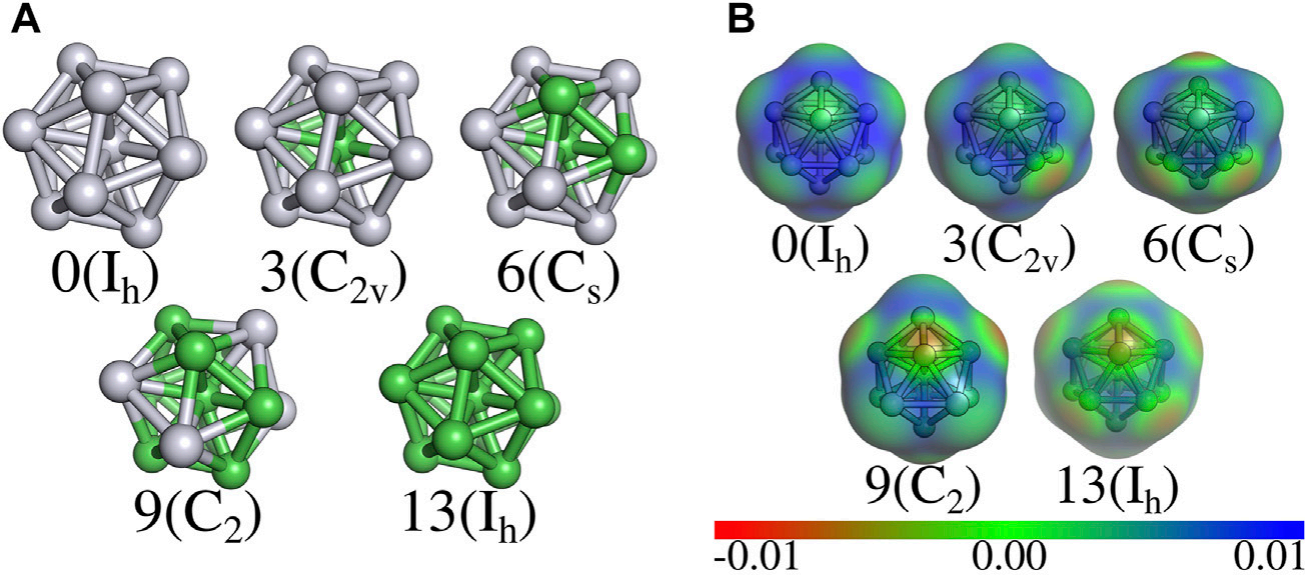
Optimized structures of the Pt_13−n_Ni_n_ (n = 0, 3, 6, 9, 13) nanoclusters **(A)** and electrostatic potential surface (EPS), in a. u, of the optimized structures **(B)**. Number of Ni atoms shown below each nanocluster structure along with the group symmetry in parenthesis. The icosahedral shape is kept for n = 0 and n = 13, as for n = 3, 6, and 9 Ni is conserved as the central atom. For n = 9 there are two Pt pair of atoms islanding at opposite nanocluster corners. For n = 3, 6, 9, and 13 an increasing number of red spots on the EPS can be observed as consequence of Ni doping process. Light gray and green colors represent platinum and nickel atoms, respectively.

Figure 2 shows the relaxed nanoclusters and the corresponding electrostatic potential surface (EPS), respectively, where the number of Ni atoms is shown along with the related symmetry group. It is important to notice that the chosen structures under the GFN-xTB, which went to the DFT-D3BJ optimization, were all Ni-centered. Since only the lowest energy structures were selected, it is interesting that a low level of optimization sampled the Ni-centered structures in accordance with a previous study showing that those nanoclusters show lower energy than those Pt-centered (Zhao et al., 2013). The stability of the nanoclusters can be quantified directly from the values of *E*_b_ and *E*_exc_. The former shows the binding strength among the atoms within the structure, and low values indicate that the atoms are bound strongly. The *E*_exc_ measures the energy associated with the alloying process, and low values imply more stable structures.

As shown in Table 1, the Ni doping process increases the value of E_b_ from -3.26 eV to -2.81 eV; as for the alloying stability, the most stable structure is reached with Pt_7_Ni_6_ with -0.1961 eV. Yet, for the average atomic distances, there is a significant increase in Pt–Pt distance for the Pt_4_Ni_9_ composition due to the islanding of the Pt atoms into two pair groups over the structure vertices (Figure 2A). Concerning the nanocluster structure, a slightly shrinking can be observed when doping with Ni as the structures with n = 3, 6, and 9, which show a constant decrease of the average atomic distances for Pt–Ni. On the other hand, the average Ni–Ni atomic distances increase from 2.35 Å to 2.43 Å. Overall, the Ni-rich structures might cause the whole system to shrink as the average atomic distance value from Pt_13_ is 2.74 Å, and 2.43 Å for Ni_13_. A consequence of the nanocluster’s shrinking is the increase of charged spots over its surface, as shown in Figure 2B. Our findings agree with previous work (Zhao et al., 2013).

**TABLE 1:** Calculated values of *E*_b_, *E*_exc_ in eV/atom and average atomic distances in Å for each nanocluster composition.

Composition	*E*_b_	*E*_exc_	D_PtPt_	D_PtNi_	D_NiNi_
Pt_13_	−3.26	0.000 0	2.74	—	—
Pt_10_Ni_3_	−3.32	−0.167 7	2.72	2.58	2.35
Pt_7_Ni_6_	−3.24	−0.196 1	2.73	2.57	2.41
Pt_4_Ni_9_	−3.11	−0.164 0	2.81	2.56	2.42
Ni_13_	−2.81	0.000 0	—	—	2.43

### 3.2 Electronic Analysis

**FIGURE 3:**
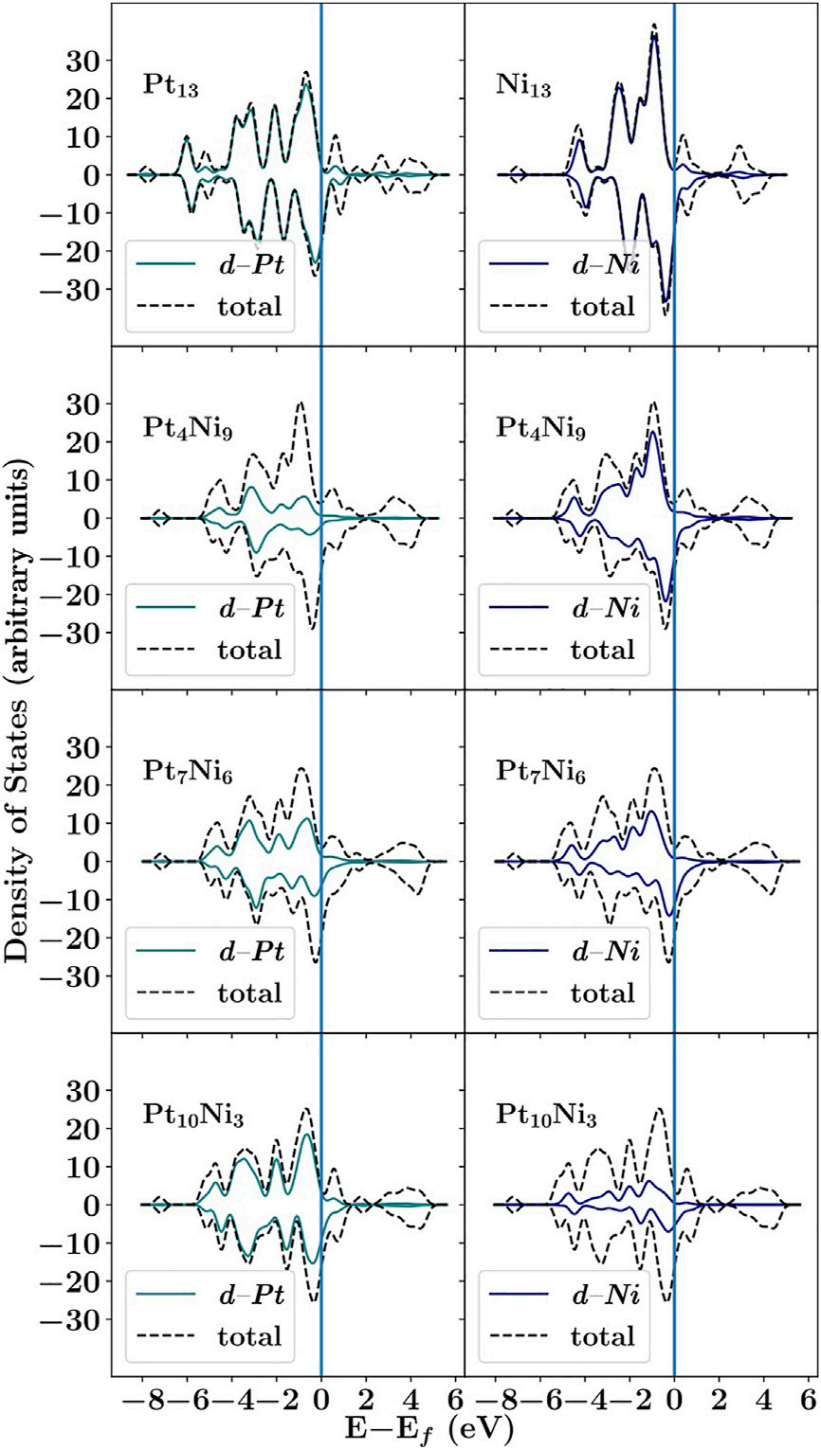
PDOS for each nanocluster composition. Pt and Ni *d*-state component is shown at the left and the right side, respectively.

The literature pointed out that Pt_13−n_Ni_n_ nanoclusters have a stronger *d*-band coupling between Pt-5*d* and Ni-3*d* as the Ni content increases (Zhao et al., 2013). Hence, it is helpful to analyze the PDOS for the metal *d*-states to understand the nanocluster stability from an electronic perspective. As presented in Figure 3, for the nanoclusters with n ranging from 3 to 9, the resemblance of the Pt-5*d* and Ni-3*d* reaches a matching profile at n = 6. Nevertheless, for n = 3 and 9, there is a higher contribution from Pt and Ni to the overall DOS profile, respectively. Therefore, a weaker coupling between the Pt and Ni *d*-states is expected for n = 3 and 9, following a higher value of *E*_exc_. In addition, an analysis of PDOS renders the presence of anisotropic spin-up (*α*) and spin-down (*β*) channels. This result suggests that a magnetic character for all nanocluster compositions could come from this misalignment because a noticeable shift towards a more magnetic character appears as a result of Ni doping: the *β* channel moves towards the Fermi level (*E*_*f*_), which might cause a decrease in the *β* channel electronic population when compared to the channel as shown in Table 2.

**TABLE 2:** Electronic population for each spin channel of the *d*-band averaged by atom for each Pt_13−n_Ni_n_ (n = 0, 3, 6, 9, 13) nanocluster.

Composition	*α*	*β*	Total
Pt_13_	4.37	4.00	8.37
Pt_10_Ni_3_	4.34	3.87	8.21
Pt_7_Ni_6_	4.30	3.64	7.94
Pt_4_Ni_9_	4.29	3.86	8.15
Ni_13_	4.45	3.98	8.42

It is already known that Pt has significant magnetic nature (Paola et al., 2017; Albert and Rubin, 1964; Zhao et al., 2013; Du et al., 2014; Wang and Johnson, 2007). Also, experiments employing superconducting quantum interference device and x-ray magnetic circular dichroism have shown a magnetic behavior for Pt_13_ when supported inside zeolite pores (Roduner, 2014; Roduner and Jensen, 2015; Liu et al., 2006; Bartolomé et al., 2009), although these systems may differ from the perspective of the free nanoclusters in vacuum as discussed in the literature (Paola et al., 2017). Nonetheless, it is also reported that other metal nanoclusters from the third transition series exhibit magnetic character at a low size regime (Yamamoto et al., 2003; Roduner, 2014). Indeed, an analysis of the results shown in Table 3 renders that our computed values for the total magnetic moment (m_T_) are in close agreement with reported values in the literature (Bartolomé et al., 2009; Zhao et al., 2013; Du et al., 2014; Paola et al., 2017).

**TABLE 3:** Calculated total magnetic moment (m_T_) in *μ*_*β*_.

Composition	*m*_*T*_
Pt_13_	2.86^a^, 2.29^b^, 2.00^c^, 3.7^d^, (2.0, 3.9, 2.34)^e^
Pt_10_Ni_3_	5.35^a^, 5.77^b^
Pt_7_Ni_6_	7.81^a^, 7.80^b^
Pt_4_Ni_9_	8.47^a^, 8.01^b^
Ni_13_	8.25^a^, 8.07^b^

a This work.

b (Zhao et al., 2013).

c (Du et al., 2014).

d (Bartolomé et al., 2009).

e computed values for vacuum, adsorbed in zeolite without D3 correction, and with D3 correction respectively (Paola et al., 2017).

Despite the magnetic behavior of those nanoclusters, the total electronic population for the metal *d*-states is roughly conserved. As proposed by the HN model, the conservation of electronic population causes a narrowing of *d*-bandwidth $\left(\bar{w}\right)$ to keep a constant number of states. In other words, as the *d*-band center (*ϵ*_*d*_) upshifts towards the Fermi level, the number of states is kept constant with shrinking (Kitchin et al., 2004). Furthermore, as the *ϵ*_*d*_ gets closer to the Fermi level, fewer states will be available to be filled. In fact, as Ni electronic distribution is 3*d* ^8^ 4*s* ^2^, fewer *β* electrons will be available in the nanoclusters’ *d*-band as Ni content increases, differently from Pt 5*d* ^9^ 6*s* ^1^. These results are in agreement with the calculated *d*-band center according to the HN (*ϵ*_*d*_), corrected HN $\left(\epsilon_{d}^{\bar{w}}\right)$ , and $\epsilon_{d}^{eff}$ methods as shown in Table 4.

**TABLE 4:** Computed values in eV of *d*-band center (*ϵ*_*d*_), bandwidth $\left(\bar{w}\right)$ , corrected *d*-band center $\left(\epsilon_{d}^{\bar{w}}\right)$ , and effective *d*-band center $\left(\epsilon_{d}^{eff}\right)$ .

Composition	*ϵ*_*d*_	$\bar{w}$	$\epsilon_{d}^{\bar{w}}$	$\epsilon_{d}^{eff}$
Pt_13_	−2.07	2.72	−0.71	−2.08
Pt_10_Ni_3_	−1.93	2.59	−0.63	−1.94
Pt_7_Ni_6_	−1.77	2.48	−0.53	−1.81
Pt_4_Ni_9_	−1.70	2.30	−0.55	−1.72
Ni_13_	−1.45	1.89	−0.50	−1.47

The changes in the electronic distribution of nanoclusters suggest that Ni doping makes the nanocluster prone to a more effective *d*-states coupling with the *σ*-states from the adsorbates. As stated by the HN model, upshifting the *ϵ*_*d*_ towards the Fermi level of the catalyst surface could favor stronger adsorption (Kitchin et al., 2004). Yet, according to Sabatier principle there is a relationship between the catalyst activity and the strength a substrate adsorbs to a surface. It states that the binding energy between the catalyst’s surface and the substrate should not be too strong or too weak (Ooka et al., 2021). Hence, as the *d*-band center suggests how strong an adsorption would occur, it can be useful for indicating which nanocluster would show a better catalytic activity.

Considering the computed values of *d*-band center in Table 4, it could be suggested that the catalytic activity for the studied nanoclusters follows the order: (1) Ni_13_ < Pt_4_Ni_9_ < Pt_7_Ni_6_ < Pt_10_Ni_3_ < Pt_13_ for both *ϵ*_*d*_ and $\epsilon_{d}^{eff}$ , but considering the value of $\epsilon_{d}^{\bar{w}}$ the order is as follows: (2) Ni_13_ < Pt_7_Ni_6_ < Pt_4_Ni_9_ < Pt_10_Ni_3_ < Pt_13_. It is also well known that clusters composed exclusively of Pt atoms present a reduced lifespan due to their tendency to CO poisoning (Ammal and Heyden, 2019; Palma et al., 2020). Therefore, the doping process with Ni atoms is expected to yield better catalytic performance for the WGS reaction. Hence, only the compositions of n = 3, 6, and 9 will be further studied herein.

### 3.3 Reaction Mechanisms

For a reaction mechanism in which CO and H_2_O will be adsorbed over a catalyst surface, a better *d*-*σ* coupling would lead to an easier activation of the reactants and a catalytic enhancement. In the following, we investigate the catalytic mechanism to determine which nanocluster composition is the favorable catalyst. By taking advantage of the RP model, we quickly assessed all the steps needed for the complete cycle of the WGS reaction through the carboxylate route (Eqs 1–8, see Figure 1). Despite the proposed mechanism shown in Eqs 1–8), steps 5 and 7 would be feasible to occur over a surface where there is enough room for the segregation process of hydrogen proton. However, in our calculations, we have observed that this step occurs in a concerted manner, in which the deprotonation process of COOH takes place as the H–H bond is formed.

**FIGURE 4:**
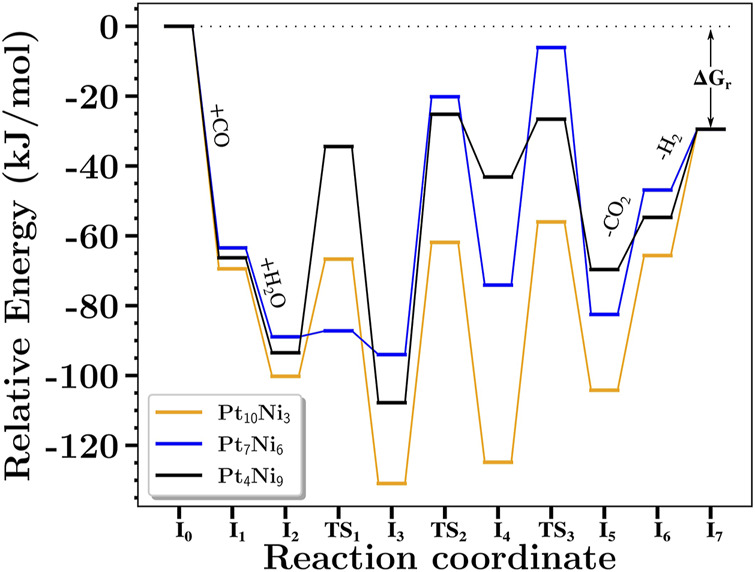
Energy profiles for the reaction mechanism of the WGS reaction.

**FIGURE 5:**
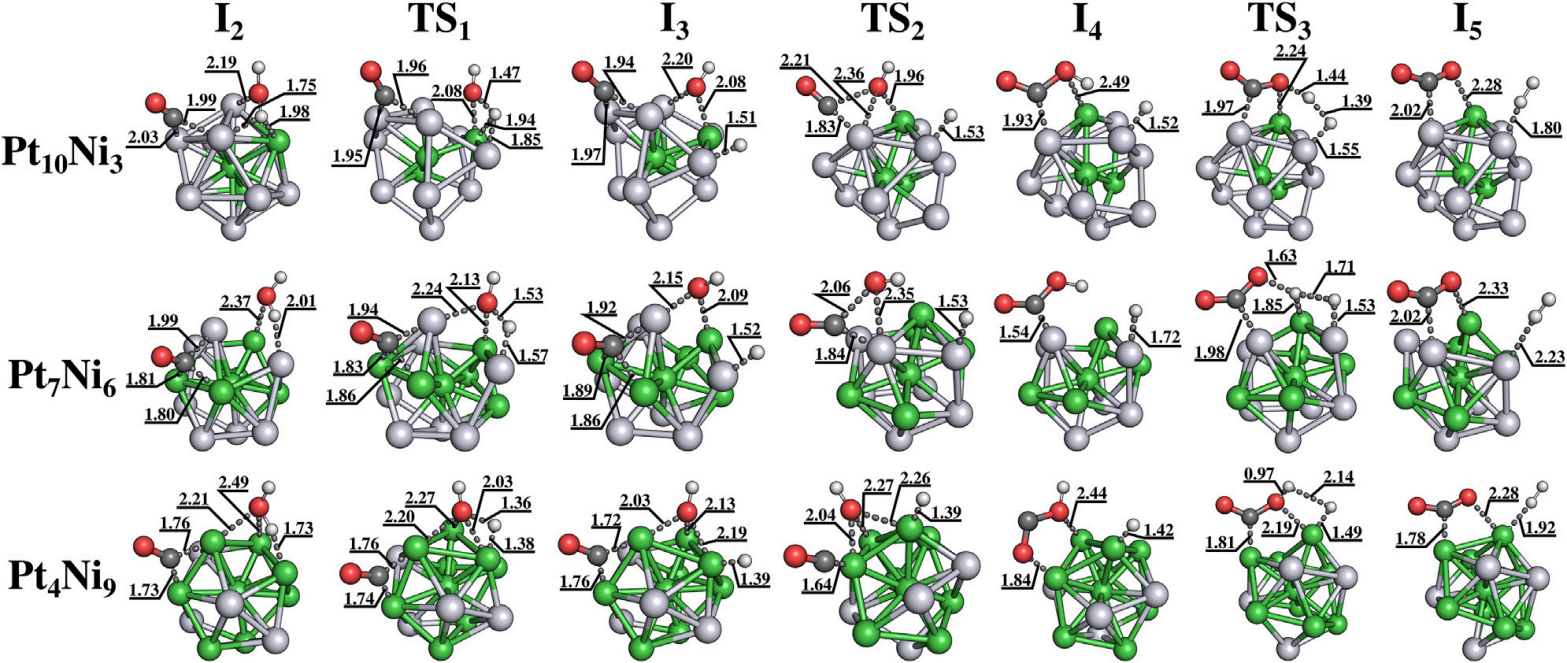
Structures of the stationary points from I_2_ to I_5_ for each nanocluster composition, Pt_13−n_Ni_n_ for n = 3, 6, and 9. The interatomic distances are given in Å. Atoms were colored in white, red, dark gray, light gray and green for representing hydrogen, oxygen, carbon, platinum and nickel, respectively.

The computed free energy profile for the complete reaction mechanism is shown in Figure 4. The steps from I_0_ to I_1_ and from I_1_ to I_2_ correspond to the adsorption processes of CO and H_2_O, respectively, while the steps I_5_ to I_6_, and from I_6_ to I_7_ correspond to the desorption processes of CO_2_ and H_2_, respectively. The WGS reaction then takes place from I_2_ to I_5_, and the transition steps are highlighted as TS_1→3_ (imaginary frequencies of TS are available in the SI). Figure 5 presents the structures from steps I_2_ to I_5_ along the values of some selected distances are also reported. As can be seen, for Pt_10_Ni_3_ and Pt_4_Ni_9_ nanoclusters, the TS_3_ step occurs in a roughly concerted manner, in which the hydrogen proton leaves the COOH as it orients itself towards the H–H formation. However, for Pt_7_Ni_6_, the hydrogen proton is slightly kept adsorbed above a Ni atom.

One key aspect to know about the catalytic mechanism is the influence that a specific step can have over the TOF values (Kozuch and Shaik, 2006). It might be possible that not only one particular pair of steps roles out the entire mechanism, thus it may be reasonable to observe the degree of control that each step has over the TOF (see Table 5). Interestingly, for n = 3 and 6, the TDTS is the TS_3_ step, but changes to TS_2_ for n = 9. As for the TDI, I_3_ is kept for all compositions. Indeed, the lowest energy step corresponds to where H_2_O has been split over the nanocluster’s surface. However, the most critical reaction step that imposes the inner catalytic energy barrier (*δ*E) for n = 3 and 6 corresponds to the proton transfer occurring at TS_3_, as for n = 9 it is the carboxylic acid formation at TS_2_.

**TABLE 5:** Degree of TOF control for each nanocluster composition within n = 3, 6, and 9. Values of energy (E) given in kJ/mol, X_TOF_ are dimensionless units.

Step	Pt_10_Ni_3_		Pt_7_Ni_6_		Pt_4_Ni_9_	
	E	X_*TOF*_	E	X_*TOF*_	E	X_*TOF*_
I_0_	0.00	−	0.00	−	0.00	−
I_1_	−69.46	−	−62.46	−	−66.28	−
I_2_	−100.22	−	−88.92	0.04	−93.49	−
TS_1_	−66.67	−	−87.21	−	−34.44	−
I_3_	−130.91	1.00	−93.99	0.96	−107.78	1.00
TS_2_	−61.88	−	−20.18	−	−25.19	0.71
I_4_	−124.83	−	−74.10	−	−43.15	−
TS_3_	−56.02	1.00	−6.09	1.00	−26.60	0.29
I_5_	−104.21	−	−82.51	−	−69.65	−
I_6_	−65.65	−	−46.88	−	−54.73	−
I_7_	−29.48	−	−29.48	−	−29.48	−

**FIGURE 6:**
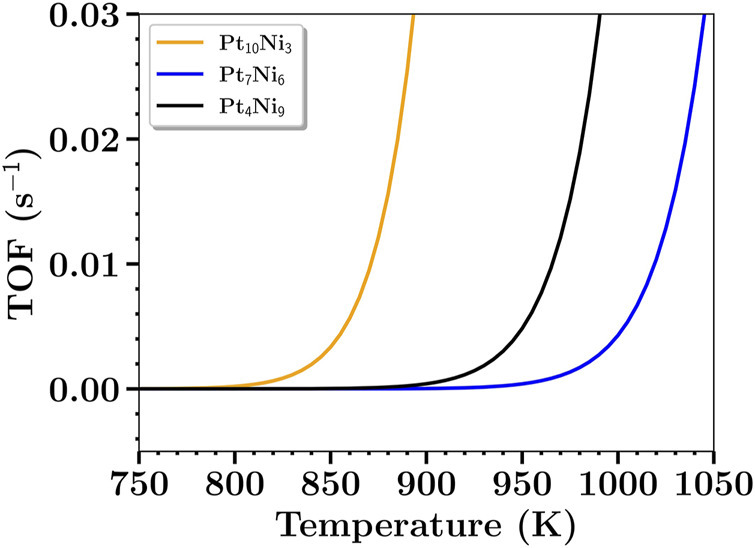
Calculated values of TOF for nanoclusters compositions of n = 3, 6, and 9 for a temperature window of 750–1050 K.

Despite those changes seen for the TDTS for n = 9, it is important to notice that TS_3_ still imposes a resistance of almost 29% to the reaction process. In other words, between TS_2_ and TS_3_, one might seek strategies to change the catalytic process to reduce the TS_2_ energy barrier to improve the catalytic process of the mechanism where Pt_4_Ni_9_ has been applied. Not only the contribution values of each TDTS and TDI for each nanocluster composition were assessed, but also the TOF value for each system under different temperatures ranging from 750 to 1050 K. As shown in Figure 6, the expected catalytic activity of the studied nanoclusters would be arranged as Pt_7_Ni_6_ < Pt_4_Ni_9_ < Pt_10_Ni_3_. From the HN model, the catalytic activity would be placed as Pt_4_Ni_9_ < Pt_7_Ni_6_ < Pt_10_Ni_3_ for *ϵ*_*d*_ and $\epsilon_{d}^{eff}$ , but Pt_7_Ni_6_ < Pt_4_Ni_9_ < Pt_10_Ni_3_ for $\epsilon_{d}^{\bar{w}}$ . Looking at the ES model description, a similar order is found for that suggested by the $\epsilon_{d}^{\bar{w}}$ model. Hence, the HN model corrected by the bandwidth seems to capture the electronic nature of nanocluster systems better.

Another important feature of a catalyst is its ability to be recovered at the end of a reaction cycle, meaning that the catalyst will return to its initial structural condition. Hence, to understand whether the nanoclusters return to their original state, we investigated the structural changes of the nanoclusters in the catalytic cycle. In that regard, Table 6 summarizes the RMSD for each nanocluster composition concerning the first step I_0_. Thus, Pt_10_Ni_3_ is more affected from structural deformations than the other nanocluster compositions. Therefore, despite the suggested good catalytic performance of Pt_10_Ni_3_ according to the ES and corrected HN models, it might not hold once a few catalytic cycles have been completed.

**TABLE 6:** Overall root-mean squared deviation (RMSD) of nanocluster structural changing for each reaction step, values given in Å.

Reference	I_2_	TS_1_	I_3_	TS_2_	I_4_	TS_3_	I_5_	I_6_	I_7_
Pt_10_Ni_3_	0.02	0.64	0.63	0.62	0.63	0.61	0.63	0.62	0.62
Pt_7_Ni_6_	0.14	0.30	0.31	0.31	0.28	0.12	0.27	0.12	0.12
Pt_4_Ni_9_	0.18	0.19	0.16	0.15	0.28	0.15	0.17	0.15	0.15

## 4 Conclusion

In the present work, a theoretical study on the catalytic mechanisms for the WGS reaction at bimetallic nanoclusters Pt_13−n_Ni_n_ (n = 3, 6, 9) is presented based on DFT calculations at the GFN-xTB level. The relative stability and structural, electronic, and magnetic properties of these nanoclusters are analyzed. The energy profile and the turnover frequency are calculated by means the reaction path and energetic span models. The main aims are the characterization of the most stable structures and putative adsorption sites on these bimetallic nanoclusters along the WGS reaction progress. The conclusions can be summarized as follows: (i) the Ni doping process provokes a slight decrease in the nanoalloys size, and a noticeable change in the magnetic property of the corresponding nanoalloy, (ii) Pt_7_Ni_6_ nanocluster is the most stable structure due to a most favorable coupling between the *d*-states coupling of Pt and Ni atoms. (iii) Pt_10_Ni_3_ nanocluster is the best catalyst followed by Pt_4_Ni_9_ and Pt_7_Ni_6_. (iv) however, despite the good catalyst performance expected from Pt_10_Ni_3_, it might not fulfill its promising catalytic behavior since a significant structural change happens after a complete reaction cycle; (v) Pt_4_Ni_9_ and Pt_7_Ni_6_ suffer a lower structural change, suggesting they would survive more cycle rounds keeping their catalytic activity, and (vi) due to its higher turnover frequency the Pt_4_Ni_9_ is expected to be a better catalyst than Pt_7_Ni_6_. We hope that the results in the current work encourage experimental studies focused on the shape and defect structure in Pt-Ni nanoalloys as catalysts for WGS reaction.

## Data Availability

The original contributions presented in the study are included in the article/Supplementary Material, further inquiries can be directed to the corresponding author.
