# Supplementary material for: A Theoretical Study on the Structural, Electronic, and Magnetic Properties of Bimetallic Pt13−nNin (N = 0, 3, 6, 9, 13) Nanoclusters to Unveil the Catalytic Mechanisms for the Water-Gas Shift Reaction
Source: Front Chem. 2022 Mar 25;10:852196. doi: 10.3389/fchem.2022.852196 (PMC9063635; doi:10.3389/fchem.2022.852196)
Supplement: Supplementary file 1 [file DataSheet1.pdf]

Supplementary Material

1 SUPPLEMENTARY DATA

1.1 Nanocluster's structure selection and analysis

For nanoclusters compositions allowed by the formula S1, we found a set of unique candidates as shown by Table S1.

$$\binom{N}{k} = \frac{N!}{k!(N-k)!} \tag{S1}$$

where N is the total number of atoms, and k is the number of doping atoms (Ni for our purposes). Since the structural generation cannot differ from symmetrical elements, a second step is conducted to identify the unique structures.

Table S1. Number of candidates for each nanocluster composition.

Composition	Candidates
Pt_{13}	1
$Pt_{10}Ni_3$	286
Pt_7Ni_6	1716
Pt_4Ni_9	715
Ni_{13}	1

The procedure adopted to identify the unique structures used a three steps approach: (1) the NP structures are read into two groups: the reference and the target; (2) each element of the target will be submitted to all possible axis swaps and reflections taken from all elements in the reference group; (3) a Hungarian reordering (Temelso et al., 2017; Allen and Rizzo, 2013; Munkres, 1957) is performed for structural alignment and only the target structure that do not show a root-mean squared deviation (RMSD) higher than 0.0001 Å was considered unique. The RMSD corresponds to a simple euclidean distance of the superimposed atoms from a given structure α and β.

For the electronic methods, the core electrons were represented with a high-quality projector augmented wave (PAW) (Corso, 2014) pseudopotential, and the valence configurations were $5d^9$, $6s^1$ and $3d^8$, $4s^2$ for Pt and Ni, respectively. Both elements were treated as open-shell systems with a starting magnetization value of 0.2 μ_β for Pt and Ni. Nonetheless, the fitted kinetic energy and density charge of the used plane-waves were set to 58 Ry and 416 Ry, respectively. For the force and energy convergences, we defined a threshold of 10^{-6} Ry/atom and 10^{-6} Ry/atom, respectively. All DFT calculations were conducted at gamma point, and the absence of imaginary frequencies assured that the structures were in their local energy minima configurations, as the presence of only one negative frequence was used to confirm the transiation structure for a given reaction mechanism.

Since the nanoclusters present a heterogeneous composition, the smearing function of choice was the Fermi-Dirac model (Mermin, 1965) along with a gaussian spreading of 0.02 Ry. Finally, for the mixing factor of the charge density, a value of 30% was chosen with local Thomas-Fermi mixing mode (Raczkowski et al., 2001). All computations were conducted with D3BJ correction (Grimme et al., 2010).

Finally, we assessed if at least three samples for each nanocluster composition had a matching energy profile as that found by GFN-xTB. In other words, we selected those candidates with the lowest, middle, and highest energy values for each composition group. The comparison can be seen in Figure S1, and it actually gives the same profile for both methods meaning that a minimal or maximal energy structure found by GFN-xTB will also matches that found by QE. We also compared the computed energy of the transition structure found by the GFN-xTB with that calculated by QE as shown in figure S2.

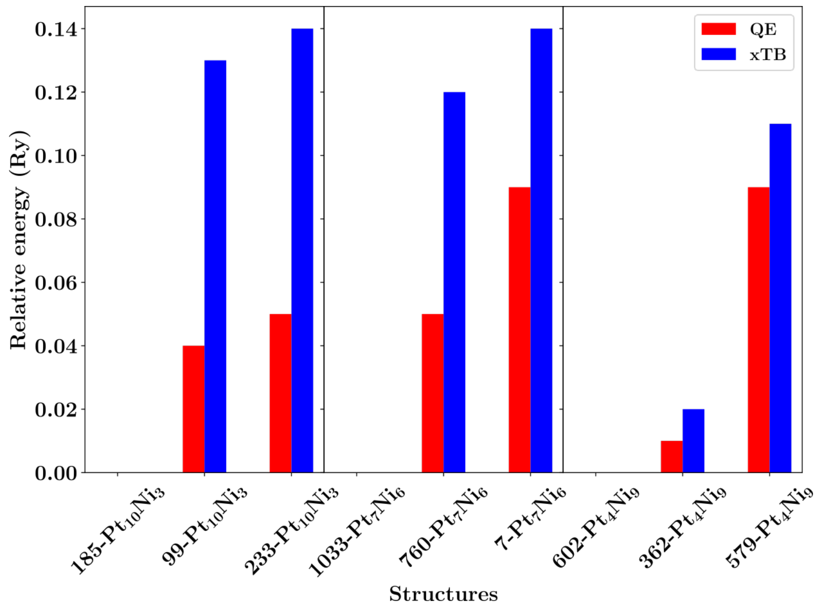

Figure S1. Relative energy comparison between relaxed structures employing QE and GFN-xTB.

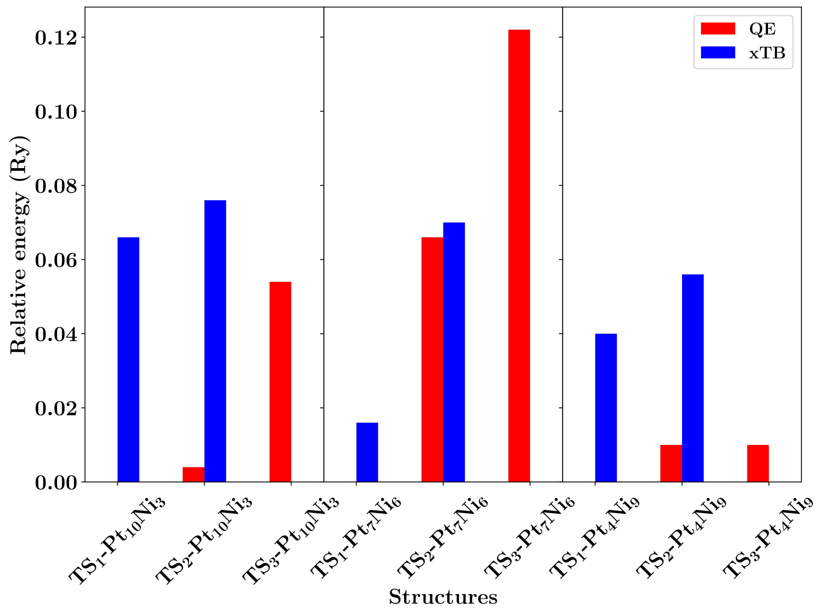

Figure S2. Relative energy comparison between transition structures employing QE and GFN-xTB.

As all computed structures had their frequencies assessed, we made sure that only hessians calculations with one negative frequency were signed as transition structures. Table S2 summarizes the negative

frequencies found for each transition structure of WGS reaciton taken for nanoclusters of $Pt_{10}Ni_3$, Pt_7Ni_6, and Pt_4Ni_9.

Table S2. Computed projected vibrational frequencies (eigval) for the transition structures, all values are given in cm^{-1}.

Composition	TS_1	TS_2	TS_3
$Pt_{10}Ni_3$	-1307.35	-281.89	-1438.29
Pt_7Ni_6	-785.46	-347.16	-971.04
Pt_4Ni_9	-1557.06	-110.98	-646.17

REFERENCES

Allen, W. J. and Rizzo, R. C. (2013). Implementation of the Hungarian algorithm to account for ligand symmetry and similarity in structure-based design. *J. Chem. Inf. Model.* 54, 518–529. doi:https://doi.org/10.1021/ci400534h

Corso, A. D. (2014). Pseudopotentials periodic table: from H to Pu. *Comput. Mater. Sci.* 95, 337–350. doi:https://doi.org/10.1016/j.commatsci.2014.07.043

Grimme, S., Antony, J., Ehrlich, S., and Krieg, H. (2010). A consistent and accurate ab initio parametrization of density functional dispersion correction (DFT-D) for the 94 elements H-Pu. *J. Chem. Phys.* 132, 154104. doi:https://doi.org/10.1063/1.3382344

Mermin, N. D. (1965). Thermal properties of the inhomogeneous electron gas. *Phys. Rev. B* 137, A1441–A1443. doi:https://doi.org/10.1103/PhysRev.137.A1441

Munkres, J. (1957). Algorithms for the assignment and transportation problems. *J. Soc. Ind. Appl. Math.* 5, 32–38

Raczkowski, D., Canning, A., and Wang, L.-W. (2001). Thomas-Fermi charge mixing for obtaining self-consistency in density functional calculations. *Phys. Rev. B* 64, 121101. doi:https://doi.org/10.1103/PhysRevB.64.121101

Temelso, B., Mabey, J. M., Kubota, T., Appiah-Padi, N., and Shields, G. C. (2017). Arbalign: a tool for optimal alignment of arbitrarily ordered isomers using the Kuhn–Munkres algorithm. *J. Chem. Inf. Model.* 57, 1045–1054. doi:https://doi.org/10.1021/acs.jcim.6b00546
